# 

**Published:** 1882-03

**Authors:** 


					﻿WH ole Number,
CCCCXLVL
March, 1882.
Number 3,
Vol. XLIV.
THE
CHICAGO
M E 1) 1CAL
T ournal and Examiner
EDITORS:
N. S. DAVIS, M.D., LL.D. JAS. NEVINS HYDE, A.M., M.D.
DANIEL R. BROWER, A.M., M.D.
CHICAGO:
PUBLISHED BY THE CHICAGO MEDICAL PRESS'ASSOCIATION,
188 Clark Street.
gyRead the Notice of this Journal among Advertisements.
				

